# Successful management of a parasitic ischiopagus conjoined twins in a low‐income setting

**DOI:** 10.1002/ccr3.1374

**Published:** 2018-01-10

**Authors:** Arlindo Rosario Muhelo, Genni Montemezzo, Liviana Da Dalt, Olivier Manzungu Wingi, Daniele Trevisanuto, Piergiorgio Gamba, Damiano Pizzol, Elena Cavaliere

**Affiliations:** ^1^ Central Hospital of Beira Beira Mozambique; ^2^ Pediatric Surgery Unit Women's and Children's Health Department University of Padova Padova Italy; ^3^ Department of Woman's and Child's Health University of Padova Padova Italy; ^4^ Operational Research Unit Doctors with Africa Beira Mozambique

**Keywords:** Conjoined twins, developing country, ischiopagus, parasitic twins, surgical separation

## Abstract

Ischiopagus parasites are fetal defects attached to a relatively normal twin by pelvis. This is the first reported case of parasitic ischiopagus twins without prenatal diagnosis successfully managed in Mozambique. A multidisciplinary team was involved in the supernumerary limbs excision. After 7 months, the infant has a normal development.

## Introduction

A parasite conjoined (or heteropagus) twin (PCT) is a grossly defective fetus, or fetal part, attached to a relatively normal twin (the autoside) 1. This is a rare anomaly with an incidence of <0.1 in 100,000 births and a frequency among all conjoined twins ranging from 4.5% to 15% 2.

Spencer's classification categorizes PCTs based on one of the eight anatomic sites in which symmetric conjoined twins are united: omphalopagus, thoracopagus, cephalopagus, ischiopagus, parapagus, craniopagus, and rachiopagus 1.

Ischiopagus parasites are attached to the autoside's lower abdomen and pelvis and account for about 13% of all heteropagus twins (54% male) 2.

These pathological conditions need a complex and multidisciplinary expertise, which is very hard to provide in a low‐income country. To our knowledge, we report for the first time a successful case of management of heteropagus ischiopagus conjoined twins in Mozambique.

## Case Presentation

A 4000‐gram girl was born to a 31‐year‐old woman, gravida 4, para 4 in a peripheral health center in the Manica province, Mozambique, after an uncomplicated term pregnancy and was referred to the Central Hospital of Beira (CHB) for a musculoskeletal malformation.

Both the mother's personal anamnesis and the family history were uneventful. The mother was HIV and syphilis negative.

**Figure 1:**
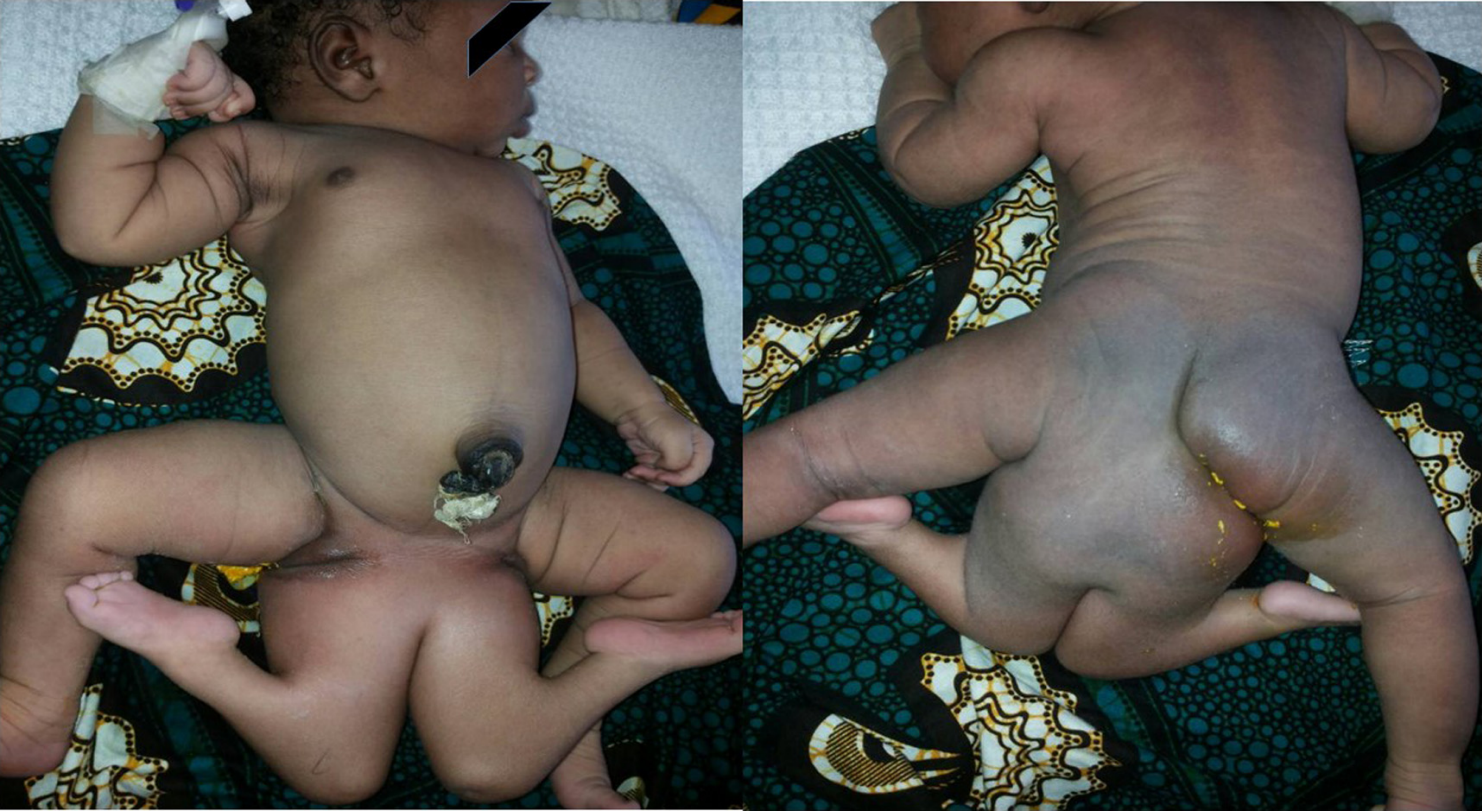
Clinical presentation of the twins when arrived at CHB (presurgical separation).

After a normal vaginal delivery, an actively crying neonate (APGAR 9‐10/10) was delivered. Physical examination revealed the presence of four limbs attached to unique pelvis (Fig. 1): Medial limbs were hypotonic, hypoplastic, cold, with no active movements and with hypo‐sphygmic peripheral pulses. Anatomically, normal toes were present in all four limbs. Female genitalia and perforated anus were lateralized to the right: a unique urethra, both labium minus, and a right labium majus were localized between the right lateral and medial limb, while the left labium majus was localized between the left lateral and medial limb.

**Figure 2:**
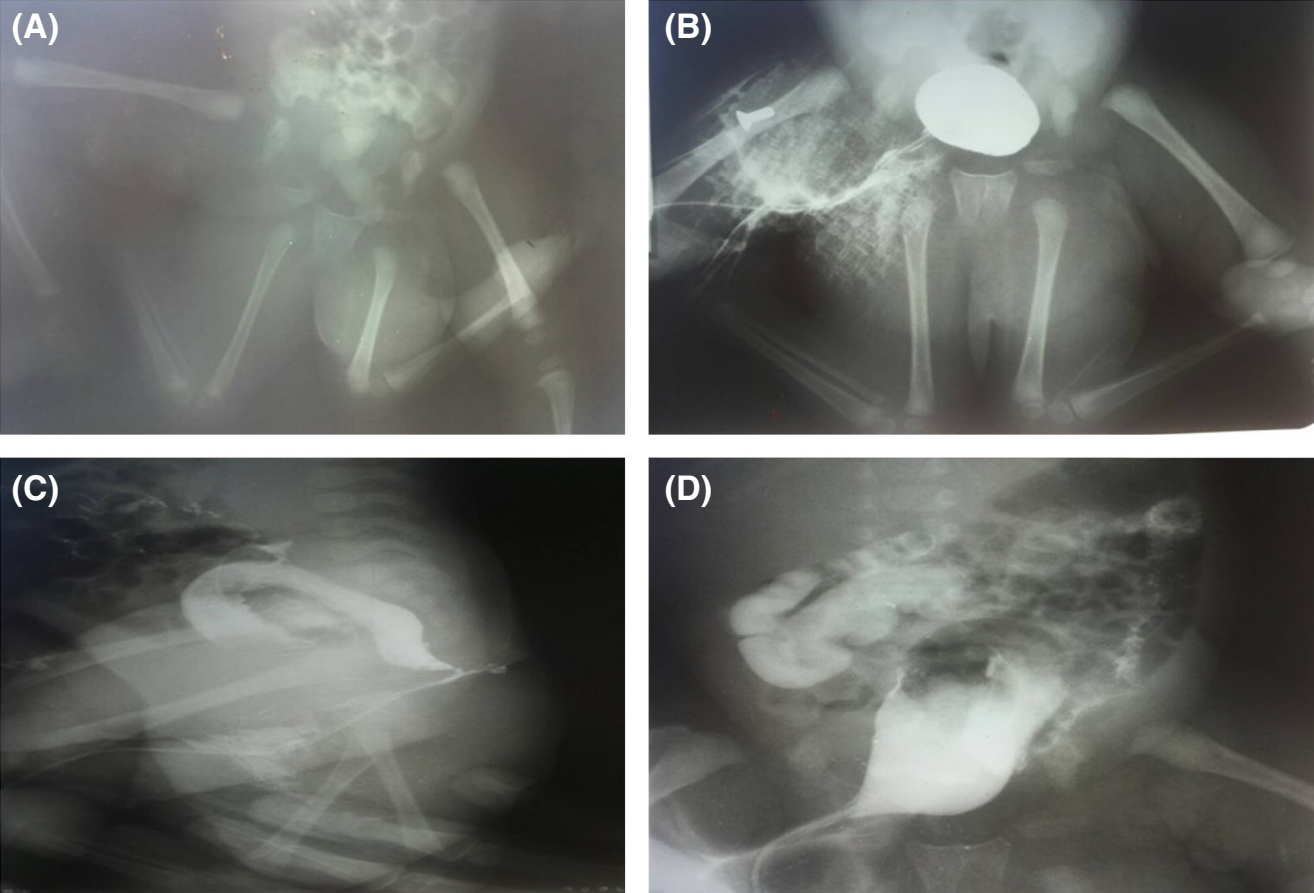
(A) Inferior limb X‐ray: four femurs, four tibias and four fibulas, one medial pair, and one distal pair each. Medial femurs were articulated with autoside's hip to its ischial part. (B) Retrograde urethrocystography: two kidneys with hydronephrosis, two ureters, anatomically normal bladder, right lateralized urethra. (C and D) Barium abdominal X‐ray: normal barium filling of the gastrointestinal tube without duplication nor dilatation or stenosis of GI tube. Right lateralization of perforated anus was evident.

Inferior limb X‐rays (Fig. 2A) showed the medial limbs were articulated to the ischial part of the hip. Barium abdominal X‐rays and retrograde urethrocystography confirmed the presence of a unique colon (with right lateralization) and of a unique bladder with only one urethra (Fig. 2B–D). Transfontanelar and cardiac ultrasounds (US) were unremarkable and two anatomically normal kidneys were evident in abdominal US.

**Figure 3:**
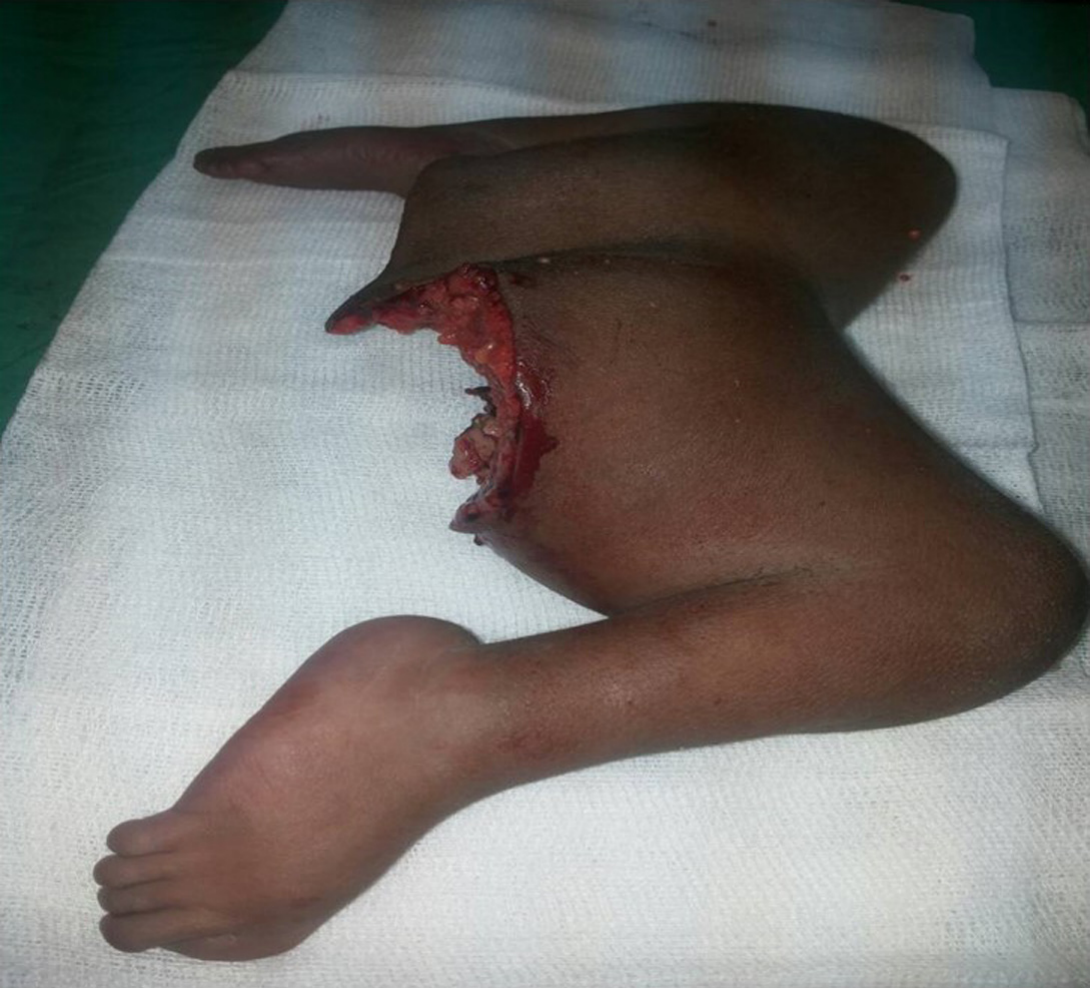
Postsurgical specimen.

The patient underwent surgery on the 16th day of life. She was placed in a lithotomic position. Vesical and anorectal catheters were positioned. In order to disjoint the medial supernumerary unfunctional limbs, a transversal sovrapubic incision was performed and this allowed a better view of the viscera. The incision was extended to the articular tissue: This allowed to see the blood supply of the supernumerary limbs which was provided by some small collateral arteries of the common femoral artery. After ligation of the arteries, disarticulation of the supernumerary medial limbs from the ischial bone was performed. During surgery, a normal right lateralized uterus and vagina were found. All specimen withdrawn were sent for histopathological examination, which, however, was not performed due to lacking of reagents (Fig. 3). The wound was closed with a median longitudinal suture, following the anatomical planes, which did not allow the reconstruction of the external genitalia. The patient was in a stationary hemodynamic condition during the 6‐h surgery, although she lost about 3 mg/L of hemoglobin. These data are consistent with similar procedures.

The postoperative recovery was characterized by late‐onset neonatal sepsis, anemia, and pyodermitis. The infant also presented a soprainfected wound dehiscence which led to a surgery cleaning for second intention after 14 days from the first intervention.

**Figure 4:**
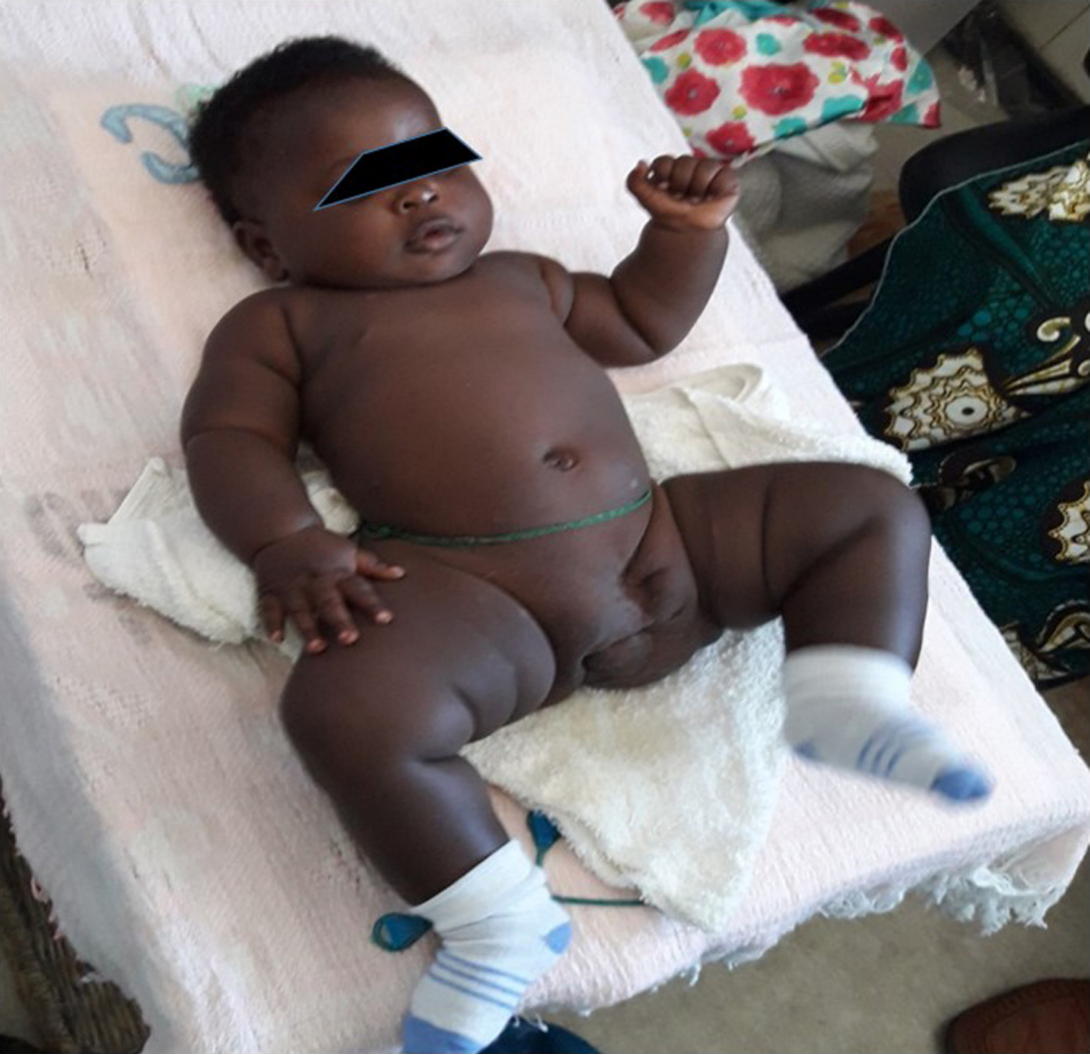
Clinical presentation at 7‐month follow‐up: She presents with a normal neurologic development: She is able to set down and to crawl. Anus and urogenital areas are lateralized to the right without any urinary or fecal incontinence.

At the age of 7 months, she is developing normally without any urinary or fecal incontinence (Fig. 4). For lateralized anus and genital area, it is planned a corrective surgery after the 12th month of life.

## Discussion

We presented a rare case of PCT in a low‐resource setting with limited means for diagnosis and management.

The duplication of inferior limbs, without any other associated malformations, allows several diagnoses: polymelia, caudal duplication syndrome associated with dipygus, human disorganization syndrome, and parasitic ischiopagus conjoined twins.

Polymelia is defined as the presence of accessory limbs attached to various body regions. This anomaly is usually associated with genetic factors and environmental agents (such as teratogenic agents), and it is very common in animals 3. It usually appears as an isolated Hypotrophyc appendix attached to the body.

The association of malformations and duplications of gastrointestinal, genitourinary systems, and neural tube defects has been called caudal duplication syndrome 4. It usually occurs with dipygus malformation and is characterized by completely duplicated legs all oriented in same direction 5.

Human disorganization syndrome is a very rare condition triggered by disorganization of morphogenetic induction resulting in anomalies of limbs (reduction or duplications, polydactyly, malformations of limb girdles), body wall defects (gastroschisis, thoracoschisis), hamartomas, and various anomalies of internal organs. Approximately two‐thirds of cases have multiple defects 6. Extremities anomalies usually involve one limb.

Finally, in typical ischiopagus PCTs, the parasite usually presents with some internal viscera 7, 8, 9, 10 and often shares genitourinary organs or colon with the autoside 1, 7, 8, 10. Moreover, it is not infrequent to find parasitic external genitalia 7, 8, 10, 11 or autoside's malformations such as defects of abdomen wall 7, 8, 9, 10 or neural tube defects 8 (Table 1).

**Table 1:** Review of literature about ischiopagus PCTs

	Year	Sex	Country	Prenatal diagnoses	Autoside anomalies	Parasite	Vascular pedicle	Shared organs	Outcome	f/u
Present case	2017	f	Mozambique	No	R lateral urethra and distal GI tract, R lateral uterus, opening vagina, L labium major separated from the rest female genitalia and located in between L lateral and medial limbs.		Femoral artery	None	Alive at last f/u	7 months
Stahr N	2015	m	Switzerland	Yes	Omphalocele, R‐side clubfoot, anal atresia without fistula, duplicated scrotum, two penises, hipospadia, bowel duplication	Kidney, external genitalia,	Internal iliac artery	Bladder	Alive at last f/u	4 months
Gokcen EC	2015	f	Ethiopia	No	Three kidneys, two bladders, two urethra, two uteri, two vaginas	External genitalia	Not identified	None	Alive at last f/u	1 year
Rode H	2006	f	South Africa	No	Only one kidney, opening cloaca	Deformed R arm, only one kidney, gross skeletal deformities, bowel in the chest, opening cloaca	Not identified	Bladder, terminal rectum, common rectal opening to the cloaca	Death after 6 days	
		m		No	Duplicated colon (rectovesical fistula R ending), anterior meningomyelocele	Male genitalia, urethra, one kidney, one bladder, one ureter, four vertebral bodies, bifid sacrum	Not identified	Anus	Alive at last f/u	5 months
		f		No		Anus, colon, blind‐end urethra, bladder	Not identified	None	Alive at last f/u	
					Not described					
Corona‐Rivera JR	2003	m	Mexico	No	Small L diaphragmatic defect, omphalocele, exstrophy of cloaca, lumbar meningocele	Complete L limb, lumbosacral vertebral column, spinal cord, one kidney with ureter and adrenal gland,	Major anastomosis	None	Death after 4 days	
Mahajan JK et al.	2002	f	India		Omphalocele, single ovary, and hemiuterus	Bowel, bladder, ovary x1, hemiuterus, external genitalia, patent urethra, BLE	Epigastric artery	Urinary (autoside ureter‐parasite bladder)	Alive at last f/u	5 months

m, male; f, female; R, right; L, left; f/u, follow‐up.

In our case, medial legs had a ventral and cranial orientation as seen in another case 9. The lateralization of distal genitourinary and rectal tracts supports the fusion theory of embryologic origin of PCT 2. The presence of parasitic viscera was not clinically or radiologically evident, but we cannot really exclude it because we did not receive any results from Pathology Service. All these data support our supposed diagnosis of parasitic ischiopagus.

This is the first case of a newborn with ischiopagus PTC managed in Mozambique. A previous case was reported in Ethiopia in a 17‐year‐old female 11. In that case, on the one hand, the older age surely led to more complex surgery, due to diminished potential of remodeling, while on the other hand, the postoperative phase surely benefitted from better recovery capacity.

In our case, a multidisciplinary team was involved (radiologists, pediatric surgeons, general surgeons, neonatologists, pediatricians, anesthesiologists) in the diagnostic phase, in order to obtain a deep preoperative study of the anatomy, at the time of surgery and during postoperative recovery. Fortunately, the absence of genitourinary or gastrointestinal tracts sharing between parasite and autoside and the lack of autoside's malformations did slightly reduce the complexity of the surgery. Moreover, great intensive care in the postsurgery recovery was made in the neonatology unit.

## Conclusion

In conclusion, we report the first ischiopagus PCT successfully managed in Mozambique. This is a rare case not only for the diagnosis, but also for the outcome, considering the low‐income setting and the lack of prenatal diagnosis, specialists, drugs, and equipment. Moreover, rapid and effective efforts are necessary in order to increase and improve health policies and win the big challenge of health equity in developing countries.

## Ethics Approval and Consent to Participate

Once the patient was admitted to the hospital, the patient's mother signed an informed consent form that allowed the medical team to be able to use the information obtained with regard to the well‐being of their child for publication. Privacy was observed at all times. Because the identities of the involved individuals are concealed, ethics approval for this particular case report was not required.

## Consent for Publication

Written informed consent was obtained from the patient's legal guardian(s) for publication of this case report and any accompanying images. A copy of the written consent is available for review by the Editor‐in‐Chief of this journal.

## Authorship

EC, ARM, DP: conceived and designed the study. EC and ARM: undertook the data collection. GM and DP: provided advice on study design and statistical analysis. EC: analyzed the data. EC and DP: drafted the manuscript with significant contributions from WMO, PG, DT, and LDD. All authors approved the final version of the manuscript.

## Conflict of Interest

The author declares that he has no competing interests.
